# Publish or Perish: Need to have another look?

**DOI:** 10.12669/pjms.322.10326

**Published:** 2016

**Authors:** Shaukat Ali Jawaid

Publish or Perish has been the driving force for the academicians, faculty members to publish and thus contribute to the medical literature besides promoting the research culture in their respective institutions. However, in view of some recent developments during the last couple of years, it has become essential to have another look on this.

Number of publication has played a vital role in the selection, appointment, promotions and approval of research grants for the faculty members and research scientists. However, now increasing number of publications from medical institutions in India, Pakistan and many other developing countries are coming out of compulsion. The main objective of the authors seems to be for selection, increments, career advancement, assessments for seeking higher qualifications like M.Phil and PhD. Now there seems to be desperation to publish and temptation to explore short cuts and easy ways. In fact it should be the quality of research and publications rather than quantity of publications which should matter.^1^

Pressure to publish is also leading to scientific fraud. Some scientists are willing to disregard scientific integrity in order to publish. For example leading publications like Nature, Science and Cell with high Impact Factors have amongst the highest rates of retraction. Peer reviewers can only study the present results but it is not always possible to detect fraudulent results. Moreover, peer reviewers can also be fooled by the fraudulent results.^2^ This is also helping the growth of predatory journals and predatory publishers. Many authors from developing countries especially India, Nigeria and some other African and Middle Eastern countries are publishing in these predatory journals. A journal which complies with ethics in publishing, which is indexed in reputed databases Indexes and like PubMed and Medline, PubMed Central, Scopus, Web of Science, is considered as reputed journals. According to reports the number of predatory journals was merely 18 in 2011 which has now increased to over seven hundred in 2015. Beal’s list also contains over twenty six misleading metrics companies fabricating spurious variants of Impact Factor. This Beal’s list provides primary guidance and information on predatory publishers, predatory stainable journals, misleading metrics companies and hijacked journals.^3^

Predatory journals promise quick publication for hard cash. Since authors in developing countries lack training, mentoring, publication pollution is increasing every day because of these predatory journals. Some of the institutions in many countries have now come up with a list of “Approved or Recognized Journals. It is worthwhile to mention here that good journals never e mail requesting authors to submit their manuscripts but for some authors, cash for easy publication is very tempting. That is why phony journals keep receiving submissions and good indexing services keep on covering them for years. Archives of Biological Sciences after detection of fraud sent the Editor along with the entire Editorial Board packing. Not only that the Serbian Ministry of Science also suspended the journal and denied it funding for two years. Management Board of Serbian Biological Society also had to resign. In this case the Editor had also published a large number of his own papers and two members of his immediate family in the journal. (4)

More recently after a series of scandals, Chinese regulators overseeing the field of academic publishing for scientific manuscripts have issued rules banning dishonest practices. The directive issued on Nov. 23^rd^ 2015 forbids Chinese Scientists from using a third party to write journal articles, sign third party to submit articles, hire a third party to substantially revise the manuscripts, providing fake peer review information or giving authorship to scientists who have not substantially contributed to the research.^5^ This action was taken by Chinese Academy of Sciences and Minister of Education after several international science journals rejected or retracted submissions from Chinese scientists because of academic dishonesty and scientific misconduct. All this raised concern about the credibility of China’s scientists and Chinese authorities. It may be mentioned here that of the 43 papers retracted by Bio Med in March 2015 following suspicions of fake peer review, 41 of these papers came from China. Earlier Sprinter had also retracted 64 articles most of them coming from China because of false peer reviews.^5^ Buying and Selling of articles has been going on in China since long because of China’s evaluation and promotion system which places an emphasis on publishing articles.^6^, ^7^

This is not all even in an Editorial on Research Integrity in China, Wei Yang wrote that Lack of research integrity may hinder China growth in original science. Not only that it will also damage Chinese academics and dampen the impact of science developed in China. He opined that action by the media to expose research misconduct, plagiarism and retractions has increased hostile public intolerance for misconduct, prompting politicians to acknowledge that a serious problem exists. Now there is more emphasis by Chinese Association for Science and Technology (CAST) and Chinese Academy of Sciences (CAS) as well as National Science Foundation of China (NSFC) to guide researchers. Major universities in China as well as CAS have revised the criteria for promotion to emphasize the quality of research contributions rather than the number of publications by a researcher. Chinese authorities now appear to be determined to achieve zero tolerance for unethical behaviour on the part of Chinese research scientists and academicians.^8^

Most authors are now keen to publish in those Journals which has got an Impact Factor. However, it is generally felt that Impact Factor is through an important yardstick to evaluate the standard of a journal but it is just one of the parameters, hence it should not be given too much importance. That was one of the reasons which forced the Academicians from various organizations and countries to issue DORA declaration i.e. San Francisco Declaration on Research Assessment a few years ago. It provides a set of recommendations regarding assessment of individuals and institutions without emphasizing the Impact Factor.^9^, ^10^ it states that while evaluating research performance focus should be given on scientific content rather than publication metrics. In addition several reputed journals like Science,^11^ Nature,^12^ British Medical Journal,^13^ Royal Society Journal of Medicine^14^ and Current Science^15^ have published articles and editorials appealing academic fraternity to take stringent and immediate measures to curb academic pollution being created by spurious/bogus predatory journals. Suggestions have also been made how to avoid predatory journals.^16^ Distinguished editors like Tom Lang while speaking at a conference on Medical Writing held at Ajman in 2015^17^ and Farrokh Habibzadeh from Shiraz former President of WAME speaking at the EMAME conference held at Shiraz in 2015 had also expressed similar views.^18^ They were of the view that the criteria for selection, promotion and heading various medical institutions should be quality of their research work in addition to their administrative and leadership qualities rather than giving importance to the number of publications alone.

Pressure to publish has also lead to many scientific frauds and misconduct. According to reports Maynooth University has revoked a former student’s PhD following investigations in circumstances which led to two previous retractions in Journal of Biological Chemistry. The university authorities conducted an investigation as per their Research Integrity Policy announced in 2014 and in accordance with the National Policy Statement on ensuring research integrity in Ireland.^19^

A University of Toronto research is also reported to have resigned over systematic data fraud. This noted Canadian endocrinologist was the lead author. She resigned from associate professorship at University of Toronto and also gave up clinical privileges at Women’s College Hospital where she was working as research director at Center for Osteoporosis and Bone Health besides division head of endocrinology and metabolism.^20^

All the above shows how concerned the regulatory authorities are in various developed and developing countries to check scientific misconduct and emphasize the importance of quality of research. Time has come that authorities in Pakistan should also give it a serious thought and come up with some practical, feasible and doable guidelines on the subject which discourages scientific misconduct and ensures that academics remain pollution free and it is the quality of research which gets importance rather than quantity.

Late Prof. Najib Khan, an eminent educationist from Pakistan often used to say that the head of a medical institution whether it is Principal of a medical college, Dean of a postgraduate medical institute or Vice Chancellor of a university is just like the Capitan of the Cricket Team. First of all his own selection and place must be assured in the final playing team based on his merit and competence. Then he or she must enjoy good moral character and his financial, intellectual integrity should be unquestionable. It is also important that he or she must be humble, easily accessible and knows the strength of each member of the team and also knows how to get best out of them in given situations. He or she must be a good listener, good at communication skills, have the courage to listen to difference of opinion from his colleagues and faculty members and assure psychological safety to those who speak out. Not only that he or she must encourage those who speak out. He or she must have leadership qualities and able to work with and lead the team to making a winning eleven. Number of publications should be just one of the parameters to be included in his/her final assessment and evaluation. These could be some of the qualities of those aspiring to occupy coveted posts like Principal, Deans and Vice Chancellors in medical institutions including Medical Universities.
